# Elucidating the crystal structure of botulinum neurotoxin type A: a personal remembrance

**DOI:** 10.1007/s00702-025-03048-1

**Published:** 2025-11-06

**Authors:** D. Borden Lacy

**Affiliations:** 1 Department of Pathology, Microbiology, and Immunology, Vanderbilt University School of Medicine, A5206F Medical Center North, 1161 21st Ave. South, Nashville, TN 37232 USA; 2 Department of Biochemistry, Vanderbilt University School of Medicine, A5206F Medical Center North, 1161 21st Ave. South, Nashville, TN 37232 USA

**Keywords:** Hans bigalke, Botulinum neurotoxin (BoNT), X-ray crystallography

## Abstract

Botulinum neurotoxins (BoNTs) are the bacterial proteins responsible for the flaccid paralysis and lethal effects of botulism. They act by inhibiting neurotransmitter release, primarily at peripheral cholinergic nerve terminals, and have traditionally been classified into seven serotypes, designated with letters A through G. Over a long history of investigation, scientists and physicians have learned to harness the selectivity and potency of these neurotoxins to advance therapeutic applications. These advances have come by leveraging the basic science understanding of how the BoNT’s work. The BoNTs are typically produced as inactive single-chain proteins of 150 kDa that can be proteolytically activated to form a 50 kDa light chain (LC) and 100 kDa heavy chain (HC) that remain linked by a disulfide bond. The BoNTs act through a multi-step mechanism in which the HC mediates receptor binding and translocation of the LC into the neuronal cell cytosol. The LC is a zinc endopeptidase and each BoNT serotype cleaves a unique and specific bond within the three-protein soluble *N*-ethylmaleimide-sensitive factor attachment protein receptor (SNARE) complex. In the late 1990s, I had the opportunity to help visualize the atomic structure of this formidable molecule, a story I share to honor the legacy of Hans Bigalke, whose work set the stage for new careers and discoveries in BoNT research and medicine.

Hans Bigalke will be remembered for his significant contributions to the understanding of botulinum neurotoxin (BoNT) mechanism of action and its development for therapeutic applications. In a series of manuscripts published from 1978 to 1981, Dr. Bigalke advanced the understanding of how BoNT and tetanus toxin (TeNT) block the release of acetylcholine at neuromuscular junctions (Bigalke et al. 1978, 1981; Habermann et al. 1980, 1981; Bigalke and Habermann 1980). His early work in adapting the phrenic nerve assay to mice (Habermann et al. 1980) led to the development of the mouse phrenic nerve hemidiaphragm (MPN) assay as a sensitive and ethical alternative to the mouse bioassay (Bigalke and Rummel 2015). In the late 1980s, Dr. Bigalke showed that neuraminidase-treated cultured cells had reduced affinity for BoNT/A and that bovine chromaffin cells lacking in polysialogangliosides became sensitive to BoNT/A when pretreated with gangliosides (Bigalke et al. 1986; Marxen and Bigalke 1989; Marxen et al. 1989). Together these studies formed the basis for realizing that gangliosides could serve as low affinity receptors for BoNTs at presynaptic membranes.

As a graduate student at UC Berkeley in 1996, I was completely unaware of these discoveries. I was an early-stage graduate student in a Chemistry department with an interest in understanding the atomic structure of proteins. The method of choice at that time was X-ray crystallography, and I had joined the laboratory of Professor Raymond Stevens, Ray, with the goal of learning how to do this. The opportunity to work on a highly potent neurotoxin that could possibly kill me was an added enticement, but that was the extent of what I knew about how it worked.

My initial project was not to look at the structure of the holotoxin though. A senior post-doc was working on that project. Ray was looking ahead and suggested that I work on the next structure, the structure of how BoNT forms a pore in the endosomal membrane. For this, my very naïve approach was to create a construct for the recombinant expression and purification of the central ‘translocation’ domain, subject the protein to low pH in the presence of detergent, and grow crystals. If only it had been that easy. While I learned some basic skills in molecular biology and working with proteins, the only crystals I managed to grow turned out to be the detergent itself (Lacy and Stevens 1997). By the time I realized the need for a new project, the senior post-doc had moved on to a different laboratory, and the task of elucidating the structure of the BoNT/A holotoxin was transferred to me. In agreeing to take on this project, I had to agree to immunization with the BoNT vaccine. This wasn’t too bad, and I enjoyed the discussion of whether I might be foregoing my future access to wrinkle reduction strategies. At the age of 24, this seemed like a minor sacrifice. Now, with the wisdom and wrinkles of age, I still would have done it. With the vaccine, I was allowed to occupy the ‘BoNT room’, a tiny, locked laboratory where we kept the active holotoxin. It was a tiny laboratory all to myself, and I was quite happy to go in there and set up crystal trays.

Here, it is important to note that the conditions for producing and crystallizing BoNT/A had already been elucidated. The protein was expressed and purified from the Hall strain of *Clostridium botulinum* type A by Bill Tepp and Bibhuti DasGupta in the Department of Food Microbiology and Toxicology at the University of Wisconsin at Madison. DasGupta, (with his permission, we referred to him solely by his last name) had started a collaboration with my mentor while Ray was finishing his post-doctoral fellowship at Harvard, and they had published a manuscript describing their first crystals in 1991 (Stevens et al. 1991). My little locked lab had a small bottle labeled as ‘Boston water’ to help address the ever-present superstitions held by crystallographers trying to reproduce crystal conditions, but in general, the protein formed large, single crystals under several conditions. The challenge for me to tackle was therefore not in getting crystals but in identifying heavy atom derivatives that could be used for solving the ‘phase problem’.

As of May 2025, there are 235,458 structures deposited in the Protein Data Bank (PDB), and algorithms like AlphaFold3 can frequently predict protein structure from the primary sequence. In 1996, there were only 4,984 structures in the PDB and no close homologs of a 150 kDa protein like BoNT/A. In the absence of a good starting model, the process of converting X-ray diffraction patterns into three-dimensional electron density maps relied on the generation of experimental phases or reference waves. This was achieved by soaking heavy atoms into the crystal. Heavy atoms have a lot of electrons and ideally bind the crystal to create subtle but detectable changes in the diffraction pattern. The idea was to collect multiple datasets on the same crystal form, one with the protein alone, one with a heavy atom soaked into the crystal, and one or more datasets with different heavy atoms soaked into the crystal and binding in different positions.

The post-doc before me had assembled a large arsenal of heavy atom compounds, many with toxicities that rivaled that of the neurotoxin. I decided to focus on compounds that wouldn’t immediately kill me if they touched my skin. I made concentrated stocks of 11 different compounds (potassium tetrachloroaurate, mercurous acetate, methyl mercuric chloride, samarium acetate, and uranyl acetate were the five that worked) and carefully transferred individual BoNT/A crystals into these solutions. I tried lots of concentrations (1-100mM depending on the compound’s solubility) and soaked for variable lengths of time (typically 6–12 h). In some cases, the compound caused the crystal to crack or dissolve, but in most cases, the crystal remained intact.

The next task was to collect small datasets with each of the soaked crystals to see if I could detect the subtle changes in the X-ray diffraction pattern conferred by heavy atom binding. This presented a new challenge - collecting a dataset before the X-ray beam destroyed the crystal. Unlike salt crystals, protein crystals have solvent channels that keep the protein hydrated and properly folded. The solvent channels serve as an entry point for the heavy atoms, but they can also contribute to rapid radiation damage as free radicals generated by the intense X-ray source move quickly through the crystal. The practice of centering crystals in a -100°C cold stream while collecting X-ray diffraction data could minimize the rapid diffusion of free radicals and preserve the integrity of the crystal. The trick was that you had to be able to cryo-cool your crystal without the formation of ice. This was typically done by soaking the crystal in a buffer containing glycerol, or anything that could serve an anti-freeze function, and then rapidly plunging the crystal into liquid nitrogen. The crystal was mounted in a nylon loop on the head of a pin and could be transferred from liquid nitrogen into the cryo-stream using metal tools that were also cooled to -100°C. If all of this happened quickly, the crystal would vitrify or ‘freeze’ without the formation of ice. However, the BoNT/A crystals were large. This was a good thing in that large crystals were needed to get a robust diffraction pattern, but it was a bad thing in that it took time for the center of the crystal to reach the same temperature as the surface. I came to realize that the act of cryo-cooling damaged the crystal integrity and made it impossible to detect the small intensity differences resulting from the introduction of heavy atoms.

After weeks of trying to overcome this problem, and in a likely act of desperation, I decided to return to the ‘old-school’ way of collecting diffraction data at room temperature. (Having never done any of this before, it wasn’t exactly ‘old-school’ but rather ‘ill-advised.‘) In the early days of crystallography, crystals were mounted in glass capillaries along with a plug of liquid to keep the crystal hydrated. The capillaries were then sealed with beeswax and could be mounted with putty on the rotating crystal mount of the X-ray generator. Using this process, I was able to identify the most promising of the heavy atom soaked crystals using our campus X-ray source.

The next step in the adventure involved going to the synchrotron. While we had the ability to collect X-ray diffraction data on campus, a synchrotron light source offered several critical advantages in terms of beam focus, intensity, and wavelength selection. At that time, there were four synchrotron light sources in the United States, with a fifth, the Advanced Light Source (ALS), being built up the hill from us at the Lawrence Berkeley National Laboratory. The beamtime was precious and whenever our lab received a slot, we would all jump at the chance to go. We therefore had many Friday all-nighters at ALS while the staff was developing workflows for general users, and I also enjoyed a weeklong trip to the Brookhaven National Laboratory in Long Island, NY. However, most of the data that went into the final structure ended up being collecting at the Stanford Synchrotron Radiation Lightsource (SSRL) in Palo Alto, CA. At each location, I got to know the beamline staff pretty quickly. Seeing someone do something as ‘ill-advised’ as shooting room temperature crystals in capillaries with a synchrotron X-ray source always drew some attention. The staff were always quite helpful though, and I knew they wanted to see me succeed. They even installed a refrigerated airjet, so that I could keep my crystals at 4 degrees while collecting data.

There is a lot that I could say about these trips. They were intense and stressful, and we got very little sleep. However, they were oddly fun, and I have many memories of the silly things we did to ensure good luck, feeling a bit loopy after eating too many chocolate-covered espresso beans with my lab mates, and us trying to help each other make good decisions with very little sleep. It was also just amazing when you put on a crystal that diffracted well. Being able to come home with a good dataset made it all worthwhile.

In most cases, we wouldn’t fully know how useful the data would be until we got back to Berkeley. While today, the computers are fast enough to complete structures at the beamlines, in the late 1990s the computers were quite slow. The computers were made even slower by the fact that we’d all come home with new data and submit our data processing jobs to the same one or two computers, essentially undermining each other’s progress with our eagerness to look at our data. Somehow, we all eventually got to the point of successfully generating electron density maps.

Having never attempted an X-ray crystallography project before, I wasn’t fully sure what to expect or look for in my first electron density maps. As it turns out, many of my initial maps were just noise, but I looked through them carefully fearing that I might miss something. In retrospect, this care was not necessary. When I finally hit upon the right combination of data and phases, the map revealed two intertwined alpha-helices, over 100 Å in length. There was no possibility of this being noise. I was there at a computer, by myself, the first-person in the world to look at the BoNT/A molecule, and it felt amazing. I have had the good fortune of many exciting discoveries in my career as a scientist, but, to this day, this moment remains one of the most exhilarating feelings I have ever experienced. I took a few hours, what seemed like a lifetime, to savor and explore those helices before sharing with Ray and my lab mates.

Of course, with the excitement, there was a new challenge, what part of the protein did the helices correspond to and where was the rest of the protein? It didn’t take long to figure out that the helices were from the translocation domain, the N-terminus of the heavy chain that was associated with forming a pore and translocating the catalytic light chain into the cytoplasm. Figuring out the rest was a giant three-dimensional jigsaw puzzle without a picture on ‘the box’, and I spent months immersed in this task. Eventually, I was able to build continuous poly-alanine chains into the densities for the LC and HC and then started the task of building in the side chains. The catalytic light chain was especially confusing though because even after assigning the whole sequence, there was a ‘belt’ of density encircling the light chain that was left over and unassigned. In fact, the belt was coming from the translocation domain and seemed to occlude access to the catalytic active site. The structure was proving to be bizarre, perhaps consistent with its unusual functions.

In today’s world it is amazing and a tad embarrassing to admit, but it took me over a year to build and refine the model that we would ultimately deposit to the PDB, 3BTA. Fortunately, the effort paid off. I was able to publish two research papers (Lacy et al. 1998; Lacy and Stevens 1999) complete my dissertation, and take a trip to Madison, WI to discuss and celebrate the structure with DasGupta and his research assistant, Bill Tepp. Ray was also very generous in letting me present the work at two conferences: the Second International Meeting on the Molecular Genetics and Pathogenesis of the Clostridia (Clostpath) in France and the 1998 Gordon Research Conference on Microbial Toxins and Pathogenesis in Andover, New Hampshire. The welcoming environments of these meetings made a significant impact on my career trajectory and my decision to remain in the toxin field.

Through conferences, I had the opportunity to meet many of the pioneering leaders of the BoNT field, including Dr. Hans Bigalke. This has always been a thrill for me as I love the stories of how science evolves. As a visual person, relying on what I see as a way of learning, I am always amazed by all that was known and understood in the absence of structure. The structure can serve as a framework for assigning mechanisms to prior observations, but usually, it helps launch a new set of questions that send us all back into the lab. In the case of BoNT, it has been incredible to see the explosion of structures (too many to cover) that describe the molecular detail of BoNT substrate and receptor specificity (Breidenbach and Brunger 2004; Benoit et al. 2014), BoNT assembly within the larger 900 kDa complex (Gu et al. 2012), and the evolutionary history of this incredible molecule (Košenina et al. 2024). With these advances, it is interesting to note that the structure of BoNT/A as a membrane spanning pore, the goal of my initial project, remains a mystery. It is possible that BoNT/A does not form a stable pore. It is likely that there is a lot left to discover about how this toxin works.

## Data Availability

No datasets were generated or analysed during the current study.
